# Mindfulness-based interventions: towards mindful clinical integration

**DOI:** 10.3389/fpsyg.2013.00194

**Published:** 2013-04-18

**Authors:** Edo Shonin, William Van Gordon, Mark D. Griffiths

**Affiliations:** ^1^Psychological Wellbeing and Mental Health Research Unit, Psychology Division, Nottingham Trent UniversityNottingham, UK; ^2^Awake to Wisdom Centre for Meditation, Mindfulness, and Psychological WellbeingNottingham, UK

## Introduction

During 2012, over 500 scientific articles on mindfulness were published. This was more than the total number of mindfulness articles published between 1980 and 2000. A recent survey by the Mental Health Foundation (MHF) found that 75% of general practitioners in the UK believe that mindfulness is beneficial for patients with mental health problems (MHF, 2010). Indeed, recent findings indicate that Mindfulness-based interventions (MBIs) may be effective treatments for a broad range of psychological disorders and somatic illnesses (e.g., Chiesa and Serretti, 2011; Fjorback et al., 2011). Given the recent growth of interest into the clinical utility of mindfulness, an appraisal of the empirical evidence and discussion of issues that impact upon the ethical standing and credibility of MBIs is timely.

## Mindfulness-based interventions

Mindfulness derives from Buddhist practice and is described in the psychological literature as an intentional and non-judgemental awareness of the present moment (Kabat-Zinn, 1990). Mindfulness is utilized in secularized interventions such as Mindfulness-Based Stress Reduction (MBSR). MBSR is a group-based program consisting of weekly meetings (~3 h duration) typically delivered over an 8-week period. The program has been delivered to over 19,000 participants since 1979 (Centre for Mindfulness, 2009). Mindfulness-Based Cognitive Therapy (MBCT) follows a similar structure (i.e., 8-weeks, group-based, weekly meetings, guided mindfulness exercises, CD for self-practice, all-day retreat) and is advocated for the treatment of specific forms of depression by the National Institute for Health and Clinical Excellence (2009) and by the American Psychiatric Association (2010). In addition to MBSR and MBCT, a number of other group-based MBIs have been developed to target specific illnesses and/or populations (Table 1). Mindfulness techniques have also been integrated into a number of one-to-one cognitive behavioral therapeutic modes such as Dialectic Behavior Therapy (Linehan, 1993) and Acceptance and Commitment Therapy (Hayes et al., 1999).

**Table 1:** **Sample of group-based MBIs along with target illness/population**.

**Mindfulness-based intervention**	**Target illness/population**
Mindfulness-based stress reduction	Various (e.g., anxiety disorders, heart disease, chronic pain, cancer, psoriasis)
Mindfulness-based cognitive therapy	Various (e.g., mood-disorders, anxiety disorders, bipolar disorder, chronic fatigue)
Mindfulness-based relapse prevention	Prevention of relapse following rehabilitation from substance-use disorders
Mindfulness-based eating awareness therapy	Binge-eating disorders
Mindfulness-based childbirth and parenting	Maternal well-being during and post pregnancy
Mindfulness-based art therapy	Psychological health and quality of life in cancer patients
Mindfulness and acceptance-based group therapy	Various psychopathologies (e.g., mood disorders, anxiety disorders)
Mindfulness-based stress management	Stress and anxiety
Mindfulness-based mental fitness training	Stress and trauma resilience for military personal

## Key strengths and supporting evidence

Meta-analytic studies of MBIs report moderately sized pre-post effects (Hedges' *g* = 0.59–0.63) for reduced anxiety and mood symptoms in study populations with somatic illnesses such as cancer, diabetes, heart disease, and chronic fatigue (Hofmann et al., 2010). However, the strongest effect sizes are typically reported for the direct treatment of anxiety and/or mood-spectrum disorders. For instance, Vollestad et al. (2012) reported effect sizes (Hedges' *g*) of 0.85–1.08 (*n* = 491 across 19 studies) for the treatment of anxiety disorders. These findings are consistent with Hofmann et al.'s (2010) larger meta-analysis who reported effect sizes of *g* = 0.95–0.97 for the treatment of both anxiety and mood-disorders (*n* = 1140 across 39 studies). Moderately sized effects have also been reported for the treatment of somatic illnesses such as chronic pain, psoriasis, heart disease, fibromyalgia, and cancer (e.g., Baer, 2003; Grossman et al., 2004).

Thus, MBIs have been shown to be efficacious for treating a broad range of health conditions and are particularly versatile in this respect. Emerging applications for MBIs include (for example) their utilization for the treatment of: (1) substance-use disorders (Witkiewitz et al., 2013), (2) problem gambling (de Lisle et al., 2012), and (3) human immunodeficiency virus (via the buffering of CD4+ lymphocyte declines; Creswell et al., 2009). MBIs also effectuate improvements in psychological well-being, cognitive functioning, and emotion regulation capacity in sub-clinical and healthy-adult populations (e.g., Chiesa et al., 2011; Eberth and Sedlmeier, 2012; Van Gordon et al., 2013).

In addition to versatility, cost-effectiveness is a further strength of MBIs as they can be delivered with as little as three facilitator hours per participant (i.e., based on 30 intervention hours delivered by one facilitator to 10 participants). Reports of adverse side-effects and relapse following MBIs are uncommon vis-à-vis psychopharmacology. Furthermore, qualitative analysis of MBI participant experiences attests to their general acceptability amongst service-users (e.g., Williams et al., 2011; Shonin et al., 2013a).

Proposed mechanisms for MBIs include: (1) perceptual re-distancing leading to increased tolerance and acceptance of somatic pain and/or maladaptive cognitive or emotional processes, (2) greater exposure to thoughts and feelings leading to reduced fear/anxiety responses, (3) greater self-awareness and self-motivation leading to improved psychosocial coping strategies, (4) reduced autonomic arousal leading to greater levels of relaxation, and (5) modification of immune and neuroendocrine system biological pathways (e.g., Baer, 2003; Ludwig and Kabat-Zinn, 2008; Compare et al., 2012).

## Limitations of MBI studies

Although the majority of MBI meta-analytic studies report moderate to strong effect sizes, such findings have not been replicated across the board. An example is Ledesma and Kumano (2009) who, based on parameters such as immunity levels, dietary fat, and hormonal indices, found only a small effect size (*d* = 0.18) for the utilization of MBIs as cancer treatments (*n* = 516 across eight studies). Irrespective of the outcome of meta-analytic studies, there are a number of methodological quality issues that hinder the wider acceptance of MBIs as mainstream psychotherapeutic interventions.

The validity of the collective findings of MBI studies is limited by heterogeneity in how different MBIs conceptualize mindfulness as well as differences in MBI program design (e.g., variations in program length, duration of weekly sessions, quantity of psycho-education, amount of physical exercise/yoga, etc.). For example, Vollestad et al.'s (2012) aforementioned meta-analysis incorporated seven different MBIs in which the total number of sessions ranged from 8 to 16, and the duration of individual sessions varied between 45 and 150 min.

Inadequate statistical power (because of small sample sizes) is a further major limitation of many MBI studies (Baer, 2003; Chiesa and Serretti, 2011). Moreover, very few MBI studies adequately control for potential confounding factors such as concurrent psychopharmacology, concomitant psychotherapy, and/or illness severity (Klainin-Yobas et al., 2012). In fact, even where a randomized controlled trial design is employed, only a small number of MBI trials could be deemed to be robust according to consolidated standards of reporting trials (CONSORT) guidelines (e.g., due to factors such as insufficient details for replication, an overall lack of transparency, absence of justification of sample sizes, etc.). Similarly, intent-to-treat analysis (ITT) is often omitted or poorly executed with limited information on how deviations from random allocation and missing data have been addressed. For example, in a recent meta-analysis by Klainin-Yobas et al. (2012), <40% of the 39 pre-post assessment studies (*n* = 1847) featured an ITT analysis, only 10% included a statistical power calculation, and ~50% of the studies were completely uncontrolled.

There is also a dearth of long-term follow-up data evaluating the maintenance effects of MBIs. For example, in meta-analytic studies by both Baer (2003) and Hofmann et al. (2010), <50% of the included studies reported follow-up data and in the case of the latter study, the median follow-up period was only 12 weeks (mean = 27 weeks; *SD* = 32 weeks). Other frequently occurring quality issues include: (1) an over-dependance in MBI studies on self-report measures, (2) fidelity of implementation not controlled for (i.e., the extent to which facilitators adhere to the delivery plan), (3) variations in the experience and competance of program facilitators, (4) participant adherance to practice data not elicited, and (5) poorly designed control interventions that do not control for non-specific factors such as total intervention hours, group interaction, and inclusion of a 1-day silent retreat component (Van Gordon et al., 2013).

## Ethical and credibility issues: an identity crisis?

Recently, Williams and Kabat-Zinn (both leading proponents in the field of MBIs), have referred to mindfulness as “*awareness itself*”, a form of “*innate capacity*” that is “*virtually transparent to us*” (2011, p. 15). The same authors also refer to secular programs such as MBSR as “*Dharma-based portals*” (“Dharma” is an explicitly Buddhist term used to refer to the teachings of the Buddha, p. 12). However, such spiritually-laden language appears to be incongruent with the general presentation and conceptualization of MBIs in relation to their operationalization within clinical settings. Thus, the identity of MBIs as well as their primary underlying “intention” (i.e., a means of improving psychosomatic well-being or a tool for spiritual development) appears to be slightly confused, and this is potentially confusing for service-users.

“Intention” underlying mindfulness practice happens to be one of the principal factors that differentiates mindfulness as taught in MBIs from its Buddhist construal. Within Buddhism, rather than psychosomatic symptom relief, mindfulness is generally practiced for the primary purpose of long-term spiritual development. In addition to what is known as “right intention” and according to the Buddhist view, mindfulness only becomes fully effective when subject to a process of cross-fertilization with numerous other practices and perspectives (Shonin et al., 2013a). Such perspectives include a profound understanding of concepts pertaining to (1) wisdom (i.e., impermanence, non-self, and suffering—known as the three Dharma “seals”), (2) meditation (including both concentrative and insight techniques), and (3) ethical awareness. These three core elements (i.e., wisdom, meditation, and ethical awareness—known in Buddhism as the “three trainings”) provide a platform for the effective development of mindfulness and are relatively undersold in the delivery of MBIs (Van Gordon et al., 2013).

Williams and Kabat-Zinn assert that rather than a “decontextualization” of mindfulness, MBIs such as MBSR execute more of a secular “recontextualization” of the Buddhist teachings in all of their “*essential fullness*” (2011, p. 15). However, and for the reasons outlined above, the accuracy of such assertions is highly questionable because even by flexible criteria, MBIs do not (and need not) represent a complete, rounded, and authentic path of Buddhist practice (secularized or otherwise). Consequently, concerns are increasingly being raised that relate to the general identity and credibility of MBIs, and the need for a unified operational approach (e.g., Rosch, 2007; Singh et al., 2008; Howells et al., 2010; McWilliams, 2011; Shonin et al., 2013b; Van Gordon et al., 2013).

Arguably the most important of these concerns are those with ethical implications for service-users. If, unbeknownst to service-users, MBIs are in fact attempting to teach Buddhism in reconstituted form within healthcare settings, then it is imperative to make this absolutely clear. Alternatively, given that MBIs claim a certain “grounding” in Buddhist philosophy, if their primary intention is geared toward improving service-user psychosomatic well-being, then there is still a need for clarity regarding what is actually implied by such a “grounding”. In other words, service-users should be made aware that mindfulness as currently operationalized in MBIs is by no means congruent with the traditional Buddhist perspective.

A further concern relates to the credibility and aptitude of MBI facilitators (Shonin et al., 2013c). Whilst referring to the stream of mindfulness teachings formulated by the likes of Kabat-Zinn (i.e., the teachings currently imparted by MBI instructors), Cullen (2011) states that MBIs are “*their own new lineage*” (p. 186). Lineage is another important concept within Buddhism and essentially refers to the “authenticity” of Buddhist teachers. In addition to receiving direct transmissions from an accomplished meditation teacher, authentic Buddhist masters generally undergo decades of focussed meditation training with the aim of relinquishing attachment to worldly concerns such as wealth, career, or renown (Shonin et al., 2013a). This is in contrast to MBI instructors who may have as little as 1 year's mindfulness experience following completion of a single 8-week course (MHF, 2010). Therefore, claims that MBIs constitute an authentic lineage in the traditional Buddhist sense are unrealistic.

## Conclusions

Interest and supporting evidence for the clinical application of MBIs has increased substantially in the last decade. MBIs appear to represent cost-effective, acceptable, and non-invasive means for treating a broad spectrum of psychological and somatic illnesses. However, future studies should address some of the methodological issues that significantly limit the validity of findings at present. More importantly, service-users are potentially exposed to oversights or misappropriations concerning the general presentation of MBIs. Whilst a certain degree of porosity between the boundary of clinical and spiritual practice does not present a problem in itself, there is a need and duty to make service-users (and the wider scientific community) fully aware of the underlying intentions of MBIs and/or of the extent to which it can realistically be said that MBIs are actually grounded in traditional Buddhist practice.
